# Experiences of capacity strengthening in sanitation and hygiene research in Africa and Asia: the SHARE Research Consortium

**DOI:** 10.1186/s12961-019-0478-2

**Published:** 2019-08-05

**Authors:** Belen Torondel, Emily Balls, Caroline Cleopatra Chisenga, Save Kumwenda, Elialilia Okello, Sheillah Simiyu, Tracy Morse, Kyla Smith, Jane Mumma, Joseph Banzi, Erik Harvey, Kondwani Chidziwisano, Jenala Chipungu, Heiner Grosskurth, Amani Beda, Saidi Kapiga, Joanna EstevesMills, Oliver Cumming, Sandy Cairncross, Roma Chilengi

**Affiliations:** 1 Department of Disease Control, London School of Hygiene and Tropical Medicine, Keppel Street, London, WC1E 7HT United Kingdom; 2 Center for Infectious Disease Research in Zambia, Lusaka, Zambia; 3 Centre for Water, Sanitation, Hygiene and Appropriate Technology Development, University of Malawi – The Polytechnic, Blantyre, Malawi; 4 Mwanza Intervention Trials Unit, Mwanza, Tanzania; 5 Great Lakes University of Kisumu, Kisumu, Kenya; 6 WaterAid, London, United Kingdom; 7 WaterAid Tanzania, Dar es Salaam, Tanzania

**Keywords:** Sanitation and hygiene programme, capacity development, consortium, training, research

## Abstract

The Sanitation and Hygiene Applied Research for Equity (SHARE) Research Programme consortium is a programme funded by the United Kingdom Department for International Development (DFID) that aims to contribute to achieving universal access to effective, sustainable, and equitable sanitation and hygiene worldwide. The capacity development component is an important pillar for this programme and different strategies were designed and implemented during the various phases of SHARE. This paper describes and reflects on the capacity-building strategies of this large multi-country research consortium, identifying lessons learnt and proposing recommendations for future global health research programmes. In the first phase, the strategy focused on increasing the capacity of individuals and institutions from low- and middle-income countries in conducting their own research. SHARE supported six PhD students and 25 MSc students, and organised a wide range of training events for different stakeholders. SHARE peer-reviewed all proposals that researchers submitted through several rounds of funding and offered external peer-review for all the reports produced under the partner’s research platforms. In the second phase, the aim was to support capacity development of a smaller number of African research institutions to move towards their independent sustainability, with a stronger focus on early and mid-career scientists within these institutions. In each institution, a Research Fellow was supported and a specific capacity development plan was jointly developed.

Strategies that yielded success were learning by doing (supporting institutions and postgraduate students on sanitation and hygiene research), providing fellowships to appoint mid-career scientists to support personal and institutional development, and supporting tailored capacity-building plans. The key lessons learnt were that research capacity-building programmes need to be driven by local initiatives tailored with support from partners. We recommend that future programmes seeking to strengthen research capacity should consider targeted strategies for individuals at early, middle and later career stages and should be sensitive to other institutional operations to support both the research and management capacities.

**Electronic supplementary material:**

The online version of this article (10.1186/s12961-019-0478-2) contains supplementary material, which is available to authorized users.

## Main Text

### Introduction of SHARE programme and capacity-building component

Basic toilets (facilities that safely separate human waste from human contact) and good hygiene practices are essential for the prevention of different diseases. A total of 2.4 million deaths could be prevented annually if everyone practised appropriate hygiene and had good, reliable sanitation and drinking water [1]. These deaths would be mostly among children in developing countries who suffer from diarrhoea and subsequent malnutrition, and from other diseases attributable to malnutrition [2]. Recent estimates suggest that, despite great progress, 2.3 billion people still lack even a basic sanitation service [3]. The dearth of this basic human right can have a profound effect on individuals’ health, wellbeing and livelihoods, and this is reflected in the priority given to “*adequate and equitable sanitation and hygiene for all*” as part of Sustainable Development Goal 6 ‘Clean water and sanitation’ [4]. Progress towards achieving universal access to this basic right is slow due to different political and economic reasons, but understanding evidence-based information of approaches and interventions that are the most effective in each setting are also crucial to achieve this progress.

The Sanitation and Hygiene Applied Research for Equity (SHARE) consortium was established in 2010 with exactly this purpose in mind – to contribute to achieving universal access to effective, sustainable and equitable sanitation and hygiene by generating, synthesising and translating evidence to improve policy and practice worldwide. The SHARE research consortium is led by the London School of Hygiene and Tropical Medicine (LSHTM) and started as a 5-year programme running from 2010 to 2015 with £10 million of funding from the United Kingdom Department of International Development. Throughout phase I, SHARE focused its activities on working closely with national sector partners from low- and middle-income countries (LMICs) to define research priorities and supported the generation of rigorous and relevant applied research. It also worked to enhance the uptake of new and existing research in accordance with the main research themes developed throughout the inception period (equity, health, markets and urban sanitation).

In late 2014, the Parliamentary Under-Secretary of State approved a £6 million cost extension, taking the programme through to December 2018. Phase II focused on four sub-Saharan African countries – Malawi, Kenya, Tanzania and Zambia. The extension was geared towards maximising the value for money of phase I, by securing the legacy and sustainability of phase I investments and furthering the research agenda in four thematic research areas in a more concerted manner (Water, Sanitation and Hygiene (WASH) and complementary food hygiene, WASH and pro-poor urban sanitation, WASH and routine immunisation, and WASH and undernutrition). Throughout phases I and II, the SHARE programme has achieved its goals with a pioneering approach that focuses on four core activities, namely sanitation and hygiene research, research-into-use, capacity development, and monitoring and evaluation.

Capacity-building has been an important and solid component responsible for the success of the SHARE programme. The gap in research capacity strengthening has been widely recognised as a major barrier for development in LMICs. Although there has been remarkable progress over the past two decades, it has been said that “*research capacity in the South remains one of the world’s unmet challenges*” [5].

The capacity-building goal of the SHARE programme was to strengthen the capacity to sustain global sanitation and hygiene research by LMIC researchers and institutions. The research capacity-building activities of SHARE were aimed at maximising individual and institutional research capacity development by using different strategies. As such, the consortium worked with its research partners and other key sector stakeholders to support them in conducting research, interpreting research findings and applying these to their work. The aim of this paper is to summarise the capacity-building strategies conducted in this programme during phase I and II and highlight the lessons learnt that could be useful for future programmes.

### Capacity-building during SHARE phase I

SHARE’s capacity development activities focused on increasing the capacity of individuals and institutions to convene stakeholders, to conduct relevant and rigorous research, and to use evidence to inform hygiene and sanitation programmes and policies. The SHARE consortium’s approach to capacity-building involved strategically designing research projects to build capacity within collaborating organisations. This was achieved through action research with advisory support from LSHTM, WaterAid, the International Centre for Diarrhoeal Disease Research, Bangladesh (ICDDR,B) and the International Institute for Environment and Development for collaborating partners.

In terms of specific training activities, the approach to capacity-building and strengthening comprised (1) structured mentoring integrated into the research, administration, financial management and communication activities; (2) specific training to address immediate gaps in skills; and (3) a PhD programme designed to build lasting research capacity within LMIC institutions (including non-governmental organisations and universities).

The structured mentoring has been an on-going activity in support of proposal development. To further strengthen the consortium’s research capacity, we included external peer review of the research protocols by independent researchers not involved in the proposal development. This critical review helped researchers in refining and shaping their research proposals. Examples of other forms of mentoring included advisory consortium members supporting in-country partners in areas of training, project costing, financial management and procedures. During this period, SHARE encouraged the different research groups to have a balanced authorship contribution of authors from high-income country and LMIC institutions in publications resulting from their work. One strategy used was to request that each research group had to prepare a list of publications before the start of each project with a discussed and agreed authorship contribution.

SHARE funded six PhD students who were selected based on the quality of their suggested research and experience in research in their countries of origin (India, Malawi, Bangladesh, Kenya, Ghana and Nepal). The topics of research selected by the students were all related to sanitation and hygiene (described in Table 1) and mentors from LSHTM were selected to direct or co-direct their Theses. All the students completed their PhD within the duration of the programme and, so far, have published 17 open access peer-reviewed papers from their work. Another output is that, since then, SHARE phase I PhD students have formed networks among themselves in the WASH sector, which has resulted in different collaborations on new projects, an idea birthed during the London PhD training period.

**Table 1:** Description of PhD student projects funded in SHARE phase I

PhD student	Title	Country
Dr Richard Chunga	Modelling household sanitation technology choices in peri-urban areas in Blantyre and Lilongwe, Malawi: a revealed preference approach	Malawi [6, 7]
Dr Parimita Routray	Gender and sanitation in Odisha, India: implications for intervention strategies	India [8–10]
Dr Sheillah Simiyu	Socio-economic dynamics of sanitation in informal settlements of Kisumu city, Kenya	Kenya [11–16]
Dr Tarique Huda	Role of sanitation in preventing contamination of the domestic environment and protecting health, Bangladesh	Bangladesh [17]
Dr Prince Antwi-Agyei	Wastewater in use in urban agriculture in Ghana: an exposure and risk assessment in Accra, Ghana	Ghana [18–22]
Dr Om Prasad Gautam	Food hygiene intervention to improve food hygiene behaviours, reduce food contamination and diarrhoeal disease burden in Nepal	Nepal [23]

SHARE also supported 25 MSc students and facilitated 26 training courses and 52 knowledge-sharing events. It provided peer review for 48 research proposals submitted to different funding rounds and offered external peer review for the national platforms’ research outputs.

### Capacity-building during SHARE phase II

Phase II constituted a transition from phase I and aimed to increase SHARE’s focus on building capacity in the target countries – Tanzania, Malawi, Kenya and Zambia. More specifically, phase II aimed to develop sector capacity with the view to ensuring the legacy of SHARE investments. In practice, this has required a transition away from investing in PhDs and MScs towards more established mid-career scientists from within SHARE partner institutions. This move aims to enhance the retention of skills and knowledge within leading national WASH institutions while also maximising the reach of investment – mid-career scientists themselves offer capacity development and support to PhD and MSc students.

In order to achieve this purpose, two main strategies were used, namely (1) a Research Fellow was appointed and supported for 24 months in each of the four targeted institutions (£52,500) and (2) all four national research partners were awarded £40,000 to implement their own capacity development plans.

#### Research Fellows

The four Research Fellows have been involved directly in their institution’s research programme, whilst also being encouraged to develop their own research agenda. A specific terms-of-reference document was designed for each of them with the aim that they would mobilise resources for sustaining their position. Each fellow had a personal development plan, to the value of £6000, that could be utilised for training, attending conferences and network meetings, among others. All the Research Fellows have been participating actively in their research programmes and have been helping to develop the capacity of other students and research staff within their institutions. All of them have been actively involved in developing their careers and in participating in workshops and conferences inside and outside their countries. In Table 2, we summarise key activities involved and show specific examples of where they have been working to ensure the sustainability of their positions within their institutions.

**Table 2:** Research fellow activities and contributions

Institution	Contributions to research project	Training organised	Training received/conference attendance	Funding applications
GLUK	- Coordinating formative stage of the main project - Coordinating the main project intervention - Contributing to research into use through stakeholder meetings - Leading the authorship of two manuscripts and contributing to other manuscripts from the study	- Reference management training to PhD students - Training on poster development and qualitative data analysis to Master’s students - Training of research assistants in methods of field data collection - Mentorship to PhD and MSc students and supervising their theses/dissertations - Lecturing Master’s and undergraduate students a course on basic research methods	- Training on transdisciplinary research (Uganda, Aug 2017) - GLUK annual conference (November 2017) - Africa Science Leadership training (South Africa, March 2018), organised by the University of Pretoria - Seedbeds of transformation conference (May 2018, South Africa), organised by Future Earth - Participant and panel discussant in the Sustainable African Cities Conference (July 2018, Ghana) - Participated in the WEDC conference (July 2018) - Participated in the International Conference on Urban Health (Nov 2018, Uganda)	- Co-principal investigator on a study on shared sanitation in Kenya and Ghana, funded by SIDA through the International Science Council - Co-principal investigator on the ‘Market to Mouth study’, a 1-year research study in collaboration with University of Iowa and IFPRI, funded by IFPRI - Study on faecal waste emptying services in Kisumu, funded by WSUP - Three other applications were made but were unsuccessful
MITU	- Leading the qualitative research component of the Mikono Safi Intervention - Contributed to the ongoing parental engagement activities in intervention schools - Lead on a formative research to develop methods to be used to measure mobility among women at high risk of HIV infection in fishing communities along Lake Victoria - Leading the authorship of two publications and contributing to other manuscripts from the study	- Co-facilitated a capacity-building session on the role of formative research in WASH in a WASH stakeholders’ meeting - Co-facilitated a research methods course conducted annually by MITU - Provided qualitative methods training to junior research scientists participating in Mikono Safi and other projects at MITU	- Learning new methods of analysis by contributing to the analysis of qualitative data from the Women’s Sanitation Vulnerabilities in Southern Tanzania, a funded SHARE project based in Iringa region - Learning how to develop measures of mobility patterns among women with high risk of HIV infection in fishing communities along the Lake Victoria - Poster presentation at UNC 2018	Contributed to grant applications: - Randomised controlled trial to assess the effectiveness of antiretroviral treatment and access to services case management intervention in increasing early linkage to HIV primary care - Mixed methods study to assess reliability of using GPS trackers and mobility phones to study mobility patterns among women at high risk of HIV infection in fishing communities along the Lake Victoria (submitted to International AIDS Vaccine Initiative)
CIDRZ	- Establish assays for testing environmental enteric dysfunction in serum and stool samples - Support evaluation of rotavirus vaccine response and evaluation of factors negatively influencing vaccine uptake - Profiled pathogens responsible for the aetiology of diarrhoea post-rotavirus vaccine introduction - Leading the authorship of two publications and contributing to other manuscripts from the study	- Provide mentorship to three PhD students and one MSc student - Participated at CIDRZ weekly research meetings - Presentation to institution research team on all available grants	- Learnt how to design and implement a clinical trial - Learnt project management skills (currently managing three projects) - Learnt grant writing skills - Attended the roundtable SHARE meeting in Geneva - Attended a EDCTP meeting in Johannesburg - Attended the grant and manuscript writing at CAPRISA, Johannesburg - Attended to in house Stata training within CIDRZ	- Received an EDCTP grant (Career Development award) of 36 months duration, aiming to determine immunogenicity against the newly introduced cholera vaccine in a Zambian population - Submitted grant applications to Welcome trust Career Fellowship Awards, Future Leaders – African Independent Research Fellowships from the Royal Society of the United Kingdom
MEIRU	- Participation in the intervention development, training of research assistants and monitoring of data - Training and supervision of Group Coordinators - Coordination of small research related to main protocol - Coordinating publication development - Dissemination of research protocol, progress and results to stakeholders - Leading the authorship of one publication and contributing to other manuscripts from the study	- Manuscript writing workshop - Research methods courses (sample size determination, mix method tools, role of bias and confounding, how to conduct literature reviews, systematic reviews and meta-analyses, monitoring and evaluation, how to use different research software) - Grant proposal writing course - Integrating social science in engineering and applied science research course for staff - How get promoted through research, consultancy, teaching, university duties and outreach for staff	- Attended a research methods conference in Tanzania organised by MITU and NIMR in February 2017 - Attended Partnership for African Social Governance Research Seminar in November 2017 - Presented at the College of Medicine Research Dissemination Conference in November 2017 in Malawi and emerged best PhD presenter - Attended a postgraduate supervision course organised by the Consortium for Advanced Research Training in Africa and College of Medicine in Malawi in July 2018 - Attended a monitoring and evaluation course organised by University of Witwatersrand in Malawi in August 2018	- Applied to the African Public Health Leaders Fellowship (October 2018 to October 2019) - Applied for the 2018 Demographic Health Surveys Fellows Programme for University Faculty from Afghanistan, Cambodia, Ethiopia, Malawi, Myanmar, Nepal, South Africa, Timor-Leste, and Zimbabwe

*CAPRISA* Centre for the Aids Programme of Research in South Africa; *CIDRZ* Centre for Infectious Disease Research in Zambia; *EDCTP* European and Developing Countries Clinical Trials Partnership; *GLUK* Great Lakes University of Kisumu, Kenya; *IFPRI* International Food Policy Research Institute; *MEIRU* Malawi Epidemiology and Intervention Research Unit at the Polytechnic of the University of Malawi; *MITU* Mwanza Intervention Trials Unit, Tanzania; *NIMR* National Institute for Medical Research, Tanzania; *SIDA* Swedish International Development Cooperation Agency; *UNC* University of North Carolina at Chapel Hill; *WASH* Water, Sanitation and Hygiene; *WEDC* Water, Engineering and Development Centre; *WSUP* Water and Sanitation for the Urban Poor

In order to motivate Research Fellows to apply to different funding opportunities, a monthly rota for sharing funding opportunities was created among them. The opportunities were shared with all the SHARE members, and Research Fellows were encouraged to share within their institutions.

#### Capacity development plans

Each partner developed their own research capacity plans by identifying several small capacity gaps that needed to be addressed. All the activities mainly aligned across five topics – increase in WASH sector capacity; increase in contribution to scientific evidence; increase in dissemination and use of evidence; increase in clinical/technical or administrative skills within the institution; and improved access to technical and information technology software and tools. The capacity development manager role supported partners to organise and execute these capacity development plans. Table 3 describes the different capacity development outcomes and inputs planned by each partner.

**Table 3:** Capacity development plans

Thematic Areas		CIDRZ	GLUK	MITU	MEIRU
1. Increase in sector WASH capacity	Outcome	Build research capacity in the WASH sector in Zambia	Build research capacity in the WASH sector in Kenya	Build research capacity in the WASH sector in Tanzania	Build research capacity in the WASH sector in Malawi
	Input	1. SHARE scholar award supports field research for two Master’s students at the University of Zambia on a WASH-related subject 2. Funding to stimulate innovative WASH designs among engineering students	1. SHARE scholar award supports four grants for Masters students at GLUK to conduct WASH research 2. One PhD student to conduct research on community health volunteers and food hygiene practices	1. Host training on WASH research methods and scientific advances	1. Creation and delivery of six training courses: - Rigorous research and use of data - Data management and analysis - Monitoring and evaluation - Geographic information systems and remote sensing - Financial management of research grants - Use of RANAS model in WASH
2. Increase in contribution to scientific evidence	Outcome	CIDRZ junior staff develop scientific writing skills	Graduate students, middle level managers and policy-makers build capacity in scientific writing	Postgraduate students undertake specific WASH research	Postgraduate students undertake specific WASH research and develop skills for scientific writing
	Input	1. Host writing skills course 2. CIDRZ staff attend writing skills course	1. PhD student attended CIDRZ writing skills course 2. Organise workshop and symposiums on WASH 3. One publication by a Master’s student 4. One publication by a PhD student	1. Funding of small research projects for MSc or PhD (×2) students 2. Individual attends CIDRZ writing skills course	1. Financial support to PhD students whose research is aligned with the SHARE II Malawi protocol 2. Workshops related to publication development 3. Three grants (13 in total including those from National Budget) for Master’s students in WASH-related studies 4. Individual attended CIDRZ writing skills course
3. Increase in dissemination and use of evidence	Outcome	CIDRZ has an active Research into Use platform	GLUK disseminates research and evidence at national and international events	MITU disseminates research and evidence at national and international events	MEIRU increase sharing of knowledge in the sector
	Input	Proactive scoping of key stakeholders nationally and scheduled engagement Dissemination of research through local and international events	1. Dissemination of research through local and national stakeholder meetings 2. Participating in international conferences (World Water Forum, Stockholm, WEDC, UNC), International Conference of Urban Health	1. Participation in international conferences (41st WEDC International Conference, Nakuru, Kenya; UNC Water and Health Conference 2018, North Carolina, USA) Dissemination of research through national stakeholder meetings	1. Support for the Water and Environmental Sanitation Network for Malawi to establish a working group for research and knowledge exchange, update website to support research dissemination and development of a repository, development and delivery of podcasts, and establishment of a database for WASH research 2. Prepared and hosted two national symposia (World Toilet Day 2017 and Global Hand Washing Day 2018) as knowledge-sharing events for researchers, practitioners and policy-makers 3. Provide funding for travel and advocacy
4. Increase in clinical/technical or administrative skills within the institution	Outcome	Improve technical skills within CIDRZ	Improve skills within GLUK	Increase skills within MITU	Increase skills within WASHTED
	Input	1. Funding for an individual to attend a placement with a high-quality grant management institution 2. Support for short-term training in ODK programming for two CIDRZ Data Managers 3. Partial support for two laboratory Research Fellows to learn specific skills in South Africa	1. Funding of workshops in biostatistics, and manuscript writing 2. Financial support for training in research ethics, ODK and data management, facilitated by KEMRI 3. Support for qualitative data analysis training by KEMRI 4. Support for WASH behaviour change approaches by the team from LSHTM 5. Laboratory skills by the team from University of Iowa 6. One staff member to receive short training in research office management 7. One staff member to receive short training in grant financial management	1. Funding for research coordinator to attend two MSc modules at LSHTM 2. Admin team develop skills	1. Developed and launched WASHTED strategic plan with support from SHARE management team 2. Supported the development and management of lunch time seminars accessible to all personnel within UNIMA 3. SHARE management team provided administrative and financial training during visits to Malawi 4. SHARE management team provided mentoring to WASHTED personnel
5. Improved access to technical and information technology software and tools	Outcome	Access to institutional licenses to enable scientific capacity development	Access to institutional licenses to enable scientific capacity development	N/A	N/A
	Input	Purchase 10 EndNote licenses; 5 N-vivo licenses and five Stata licenses to be installed on institutional computers	Purchase of statistical analysis and reference management software and database		

*CIDRZ* Centre for Infectious Disease Research in Zambia; *GLUK* Great Lakes University of Kisumu, Kenya; *KEMRI* Kenya Medical Research Institute; *LSHTM* London School of Hygiene and Tropical Medicine; *MEIRU* Malawi Epidemiology and Intervention Research Unit at the Polytechnic of the University of Malawi; *MITU* Mwanza Intervention Trials Unit, Tanzania; *ODK* Open Data Kit; *RANAS* Risks, Attitudes, Norms, Abilities, and Self-regulation; *SHARE* Sanitation and Hygiene Applied Research for Equity; *UNC* University of North Carolina at Chapel Hill; *UNIMA* University of Malawi; *WASH* Water Sanitation and Hygiene; *WASHTED* Water Sanitation Health and Appropriate Technology Development, Malawi; *WEDC* Water, Engineering and Development Centre

SHARE LSHTM members and other researchers from the Environmental Health Group at LSHTM, provided continuous and responsive capacity development to all SHARE partners. This support aligned directly with partner capacity development plans and other changing needs arising during project development. Planned technical support for SHARE research partners included the following:

- *Point-of-use support:* Provided, usually remotely, by the SHARE Finance Officer, Administrator and Chief executive officer when difficulties were encountered by partners in the submission of financial, management and resources data support.
- *Mentoring:* Provided to cover specific needs, especially for senior research and management staff as well as junior academics. Mentoring was usually organised by the capacity-building manager and provided remotely by Skype or during field visits by SHARE staff or researchers from the environmental health group and responded directly to the needs of SHARE partner staff. Mentoring focused on the following topics: scientific skills such as writing academic papers or conference presentation or using referencing software, methods of analysis of scientific data, research uptake, grant management support and advice about career development.
- *Support for outcome mapping and research-into-use:* Each of the partners was supported to create outcome mapping documents to guide the implementation, monitoring and evaluation of their research-into-use work. This included identifying stakeholder outcomes across seven key groups – national government, local government international agencies, non-governmental organisations and civil society, national research institutes, donors, and research participants [24]. For each stakeholder group, partners developed specific indicators defining the desired change in stakeholder behaviour as well as generating a list of research-into-use activities to influence stakeholders. Progress was followed through quarterly tracking and reporting tools.

SHARE partners were required to report on progress against their planned capacity development activities in their quarterly reports to the monitoring officer. Data was used to measure progress towards the overarching objectives of the capacity development work stream through a programme-monitoring logframe.

### Discussion and reflections

The research capacity-building activities of SHARE were aimed at maximising individual and institutional research capacity development by using different strategies. The main strategies that yielded success were learning by doing (supporting institutions and postgraduate students on sanitation and hygiene research with different activities), providing fellowships to appoint mid-career scientists to support personal and institutional development, and supporting tailored capacity-building plans.

In phase I, SHARE’s capacity development activities focused on increasing the capacity of individuals and institutions, whilst phase II constituted a transition from phase I and aimed to increase SHARE’s focus on building capacity in higher research and education institutions from four target countries to ensure the legacy of SHARE. There was a clear transition from investing in PhD and MSc to more established middle-career scientists, with a specific budget invested in retention of skills and knowledge within leading partners institutions. A key goal for sustainability of any African institution is to ensure that research staff are advancing in their career progression and consolidate their research skills. Barriers to this, such as poor funding and lack of protected time for research pursuits, have been a common complaint from African researchers [25], which is why the Research Fellows scheme was a key success of the SHARE programme. Investing in postgraduate and doctoral studies in phases I and II was also an important achievement of the SHARE programme, as the need to provide more support for postgraduate training in health sciences in African universities is globally recognised [26, 27] if we are to increase the capacity of institutions. In both phases, locally driven research agendas were a priority. It has been seen from other programmes that externally dictated agendas have resulted in inappropriate projects unrelated to local research needs, which derived conclusions that did not have any direct local benefit [28, 29].

The SHARE consortium has managed to meet most, if not all, of its goals in the complex field of WASH. All phase I projects were completed and phase II projects are on their way to completion. The consortium projects have generated, synthesised and tested practice on current critical issues in the WASH sector. In the first phase, this included sanitation technology assessments; understanding gender and sanitation issues; the socioeconomic dynamics of sanitation to water use; and food hygiene in various contexts of LMIC settings. The second phase saw the consortium focus on supporting work on WASH and complementary food hygiene; WASH and pro-poor urban sanitation; WASH and routine immunisation; and WASH and undernutrition. Through these projects, a wealth of experience has been developed, with 114 peer-reviewed manuscripts published in international journals (Additional file 1: Table S1). Deliberate strategies were applied to distribute authorships between members of high-income countries and LMIC partner institutions; these achieved a good balance of authorship, in contrast with other programmes where power-imbalanced relationships resulted in more published work led by high-income country researchers [28]. Perhaps most importantly, SHARE will leave a legacy through its support for sustainable capacity development that will live on decades after the consortium is closed. Formation of a peer network of researchers was the pivot of success of the SHARE programme – as a result nine networks were created during phase II of the project (Additional file 2: Table S2). At the end of this 8-year programme, SHARE has effectively managed to roll out the capacity-building programmes for these nine networks (Tables 2 and 3), all aiming to orientate early- and middle-career researchers towards WASH research and equipping them with the necessary skill-set needed to carry out scientific research.

The capacity-building strategies from SHARE have been successful at meeting programme goals because of three important factors that were considered during the programme. First, strategic national partner selection – the partners selected within Africa and Asia were all established and had the required basic systems and structures in place to allow for the projects to run effectively. Thus, the SHARE project was an addition to bolster and support work within their mandates. Second, locally driven research agendas – while the partners’ projects were invited to respond to a broad call, the research agenda was driven based on local needs and partner aspirations. Third, dedicated technical support and networking – networking activities were lined up throughout the SHARE programme. Structured milestone reporting on progress helped the projects identify areas of weakness early on and find mitigating solutions in good time.

The SHARE programme faced some administrative challenges in the implementation of its research and capacity-building activities. Different sets of administrative regulations across the institutions led to complications and delays in starting or sustaining certain capacity-building activities.

Another challenge for the appointed Research Fellows was to balance their work burden as they were involved in the programme research activities as well as the training and support for their institutions and their own development. A mitigation strategy for future programmes is to create an appropriate work plan that should be always agreed on and monitored by their line managers.

The approaches described above focus on individuals and provides relatively quick and quantifiable training outputs; these approaches serve as the backbone of human resource development for national research systems. However, without a coordinated national plan and a strong enabling environment to support trained scientists, brain drain is still likely to occur [30].

## Conclusions

Global health partnerships and international research collaborations have enormous potential to improve the WASH situation and policy in Africa and Asia. A good capacity-building component should be included in all collaboration programmes; such an approach is more likely to help the sustainability of these institutions and to generate long-term changes in policy and practice that make real and sustained improvements with regard to sanitation and hygiene issues. Trusted long-term, high-income country collaborations that understand the context and needs of the region can teach agenda-setting skills and assist in agenda development [31], but LMIC researchers should be the leaders who dictate research agendas in their countries [29], as has been the case in the SHARE programme.

### Recommendations

1. Research capacity-building activities need to be planned and tailored with each research partner institution.
2. Investments in middle-research careers are important to contribute to the strengthening of research institutions and to promote sustainability.
3. Mentoring, training and participation of postgraduate students in research projects co-led by high-income country researchers provide a great opportunity for co-learning and capacity strengthening.
4. Incorporation of networking activities in capacity-building plans offers an opportunity of future collaborations.
5. Structured milestone reporting on progress helps identify and address challenges.
6. Resource mobilisation and grant writing skills are key and must be incorporated in capacity development plans early enough in the programme.

## Additional files

Additional file 1: List of peer-reviewed manuscripts published during the SHARE programme. (XLSX 70 kb)

Additional file 2: List of researchers networks created during the SHARE programme. (XLSX 12 kb)

## Data Availability

Not applicable.

## Abbreviations

LMICs: low- and middle-income countries
LSHTM: London School of Hygiene and Tropical Medicine
SHARE: Sanitation and Hygiene Research in Africa and Asia: the SHARE Research Consortium
WASH: Water, Sanitation and Hygiene
